# Hormetic Effects of 7‐Ketocholesterol in Preventing Ferroptosis in Hepatocytes

**DOI:** 10.1155/omcl/7958511

**Published:** 2026-02-11

**Authors:** Sarit Anavi, Nicole Giltman, Haim Zeigerman, Zecharia Madar, Oren Tirosh

**Affiliations:** ^1^ Nutrition Science, Peres Academic Center, Rehovot, Israel, huji.ac.il; ^2^ Institute of Biochemistry, Food Science and Nutrition, RHS Faculty of Agriculture, Food and Environment, The Hebrew University, Rehovot, Israel, huji.ac.il

**Keywords:** cell death, lipid metabolism, oxidative stress, oxysterols

## Abstract

**Background and Aims:**

Ferroptosis, a form of cell death marked by iron dysregulation and lipid peroxidation, has been implicated in liver and neurodegenerative diseases. 7‐ketocholesterol (7KC), a cholesterol oxidation product, induces oxidative stress and inflammation at high concentrations. However, the effects of low, subtoxic levels of 7KC are unclear. This study investigates the potential hormetic effects of low concentrations of 7KC on hepatocytes and neuronal cells during ferroptosis.

**Method:**

Ferroptosis was induced in AML12 hepatocytes using 20 µM Erastin, with cells co‐treated with varying concentrations of 7KC. Cell death was assessed, and gene expression was analyzed via RNA sequencing (RNA‐seq) and real‐time PCR. HT4 hippocampal neuronal cells were treated with glutamate to induce ferroptosis, both with and without 7KC.

**Results:**

Low, nontoxic concentrations of 7KC protected both hepatocytes and neuronal cells from ferroptosis induced by Erastin and glutamate, respectively. In contrast, higher concentrations of 7KC increased cell death. 7KC’s protective effects in hepatocytes were linked to lower malondialdehyde (MDA) levels and reduced peroxidation of polyunsaturated fatty acids (PUFAs). The cholesterol synthesis pathway, as well as lipid accumulation, was suppressed by 7KC. Conversely, these processes were upregulated by Erastin. Notably, 7KC showed a stronger anti‐ferroptotic effect than 7‐dehydrocholesterol (7DHC) at low concentrations, possibly through Nrf2‐independent upregulation of the gamma‐glutamylcysteine ligase catalytic (GCLC) unit.

**Conclusion:**

This study reveals that 7KC can have a hormetic effect on ferroptosis at low concentrations, suggesting a potential advantage. Further research is needed to clarify the underlying mechanisms, particularly regarding GCLC upregulation.

## 1. Introduction

Cholesterol homeostasis and metabolism are highly regulated by the liver. In the effort to avoid cell damage due to augmented intracellular cholesterol levels, hepatocytes direct excess cholesterol for oxidation to oxysterols [1], which are essential intermediates in bile acid production. Regarding physiological role, some oxysterols are ligands for liver X receptors (LXRs). Oxidized sterols at the 22, 24, and 25 positions are potential activators of the LXR*α* nuclear receptor, while oxidized sterols at the seven position are not. LXR*α* activation by the former oxysterols can upregulate cholesterol 7*α*‐hydroxylase (CYP7A1) activity, which is the rate‐limiting enzyme in the bile acid synthesis/cholesterol removal pathway [2–4].

Oxysterols at the seven position can be generated by enzymatic and nonenzymatic processes involving enzymes of the cytochrome P450 family and reactive oxygen species (ROS), respectively [1, 5]. 7‐ketocholesterol (7KC) can be produced enzymatically via CYP7A1 metabolism as well as by ROS, and as such can be produced endogenously or be ingested from food [6]. Concerning the latter, 7KC is found in foods with high cholesterol content, especially industrial products, with levels being accelerated by intricate food production techniques, heating, air exposure, and prolonged storage [7].

7KC at elevated concentrations was shown to induce toxic effects, including the promotion of ROS generation and inflammation, cell membrane disruption, and stress response activation on different types of cells from different species [8, 9]. This oxysterol is also acknowledged for its ability to initiate cell death (often oxiapoptophagy), which is associated with apoptosis, autophagy, and oxidative stress [6, 10]. As such, the implication of 7KC in many pathological conditions and age‐related disorders has been suggested and is receiving much attention. This oxysterol is intrinsically associated with cardiovascular diseases, such as atherosclerosis [11], with accumulated levels found in arterial plaques and related to macrophage cytotoxicity via the induction of inflammation [6, 7, 12] 7KC is further implicated in several liver pathological conditions, including hepatitis following cholesterol overload, as well as exacerbating hepatic steatosis, fibrosis, and inflammation in an obese mouse model. A connection between increased 7KC levels of specific areas of the brain and neurodegenerative conditions, such as Alzheimer’s and Parkinson’s diseases, has also been suggested, given its role in oxidative stress, inflammation, as well as lipid and protein homeostasis disruption. Elevated levels were also found in other damaged tissues, including the retina, during age‐related macular degeneration [6, 7, 12]. Importantly, contrary to expectations, according to the MSDS safety data sheet of this compound, no acute or chronic toxicity data is available. Furthermore, little data are currently available regarding 7KC’s physiological function or beneficial effects. Expanding current knowledge of potential physiological functions of the 7KC may enable the utilization of this oxysterol for the establishment of new, innovative therapeutic opportunities in health and disease [13–16].

Cell death is essential in developing multicellular organisms and maintaining tissue homeostasis by depleting nonfunctional cells. It also plays a crucial role in immune responses and is associated with several diseases resulting from abnormal or defective cell death signaling [17]. First described in 2012, Dixon et al. [18] named the cell death that is induced by oxidative stress, loss of cellular thiols, and membrane oxidation, which activates a death signal, as ferroptosis. Ferroptosis is a distinct form of programed cell death that depends on iron and is manifested by elevated membrane lipid oxidation and accumulation of oxidized lipids. Ferroptotic cells have distinct features, including loss of membrane integrity, changes in mitochondrial size and morphology, and damaged outer mitochondrial membranes [19]. Ferroptosis involves the depletion of intracellular thiols due to the depletion of cysteine and reduced glutathione (GSH), the inactivation of glutathione peroxidase 4 (GPX4), which regulates membrane integrity [20], and the accumulation of iron and lipid peroxidation products. The study by Peleman et al. [21] implicated ferroptosis as a damaging factor in metabolic dysfunction–associated steatotic liver disease (MASLD) patients and implies that inhibiting ferroptosis could offer a promising therapeutic approach for such pathology. Similarly, ferroptosis has emerged as a significant contributor to neuronal degeneration and, consequently, the development of neurodegenerative diseases, such as Alzheimer’s and Parkinson’s. Mechanistically, ferroptosis is closely correlated with glutamate‐induced neuronal toxicity, where excessive glutamate release triggers mitochondrial dysfunction and oxidative stress, eventually leading to neuronal death [22]. Consistent with this notion, Xia et al. [23] demonstrated that inhibiting ferroptosis can reduce glutamate toxicity and help preserve neural integrity.

A possible connection between cholesterol and ferroptosis has been suggested [24]. This connection could be via the mevalonate (MVA) metabolic pathway [25]. Cholesterol is synthesized de novo through the MVA pathway, where *β*‐Hydroxy *β*‐methylglutaryl‐Coenzyme A (HMG‐CoA) reductase functions as the rate‐limiting enzyme [25, 26]. HMG‐CoA reductase converts HMG‐CoA to coenzyme A and l‐MVA, leading to the production of isopentenyl pyrophosphate (IPP), which is essential for GPX4 synthesis [27]. In addition, 7‐dehydrocholesterol (7DHC), a precursor of cholesterol, was recently suggested to function as a membrane radical scavenger protecting hepatocytes against ferroptosis [28]. 7KC can be generated following the oxidation of 7DHC by CYP7A1 [7]. Thus, it is surmised that 7DHC oxidation by CYP7A1 to 7KC may have a regulatory effect on cholesterol synthesis and may affect ferroptosis. In addition, 7KC has been shown to impair normal cholesterol metabolism by reducing the levels of the MVA pathway enzymes, which further suggests 7KC may influence ferroptosis via this mechanism [25, 29].

In the current study, we investigated the physiological roles of low concentrations of 7KC in ferroptosis. We hypothesize that subtoxic levels of this compound might have beneficial physiological hormetic effects that can ameliorate toxicity under lethal pro‐ferroptotic conditions.

## 2. Materials and Methods

### 2.1. Chemicals

7KC was purchased from Avanti. Green CMFDA was purchased from Cayman Chemical. 7DHC, chenodeoxy cholic acid (CdCA), dimethyl sulfoxide (DMSO), ethanol, Erastin, 1S,3R‐RSL 3 (RSL3), L‐glutamic acid, and Mevastatin were purchased from Sigma–Aldrich, Israel. Cholesterol was purchased from Holland Moran, Israel. The nuclear factor erythroid 2‐related factor 2 (NRF2) specific inhibitor ML385 was purchased from Sigma Israel.

### 2.2. Cell Culture and Treatment

The mouse highly differentiated hepatocyte cell line, AML‐12 (CRL‐2254‐ATCC), was used, as well as the mouse hippocampal HT4 neuronal cells. Cells were propagated at 37°C in 5% CO_2_ with high‐glucose Dulbecco’s modified Eagle’s medium (DMEM) supplemented with 10% FBS, penicillin (100 U/mL), and streptomycin (100 mg/mL). We used cells at passage 27 or lower for the experiments. For 24 h of treatments, cells were seeded in 6‐well plates at a density of 3 × 10^5^ cells/well. After ~24 h of seeding, the plates were subjected to various treatments, including Erastin at concentrations of 20 µM, 7KC (5–100 µM).

RSL3 and Erastin were dissolved in DMSO. 7KC, 7DHC, and cholesterol were dissolved in ethanol. L‐Glutamic acid (for the treatment of the HT4 cells, 10 mM) and chenodeoxycholic acid (CDCA) were dissolved in DDW. Control cells were treated with ethanol and DMSO.

### 2.3. Propidium Iodide Exclusion‐Cell Viability Assay

Cells were subjected to trypsinization, followed by washing with PBS and centrifugation at 1500 rpm for 4 min (Thermo Scientific, Heraeus Megafuge 16R, Osterode am Harz, Germany) at 4°C. The cells were then resuspended in PBS, filtered through a 90‐µm mesh grid, and analyzed by flow cytometry. Subsequently, the cells were stained with PI at a 0.5 µg/mL concentration. (Stratedigm, SE520EON, San Jose, CA). The analysis was performed with a flow cytometer and Cell CapTure Analysis software version 4.1 (Stratedigm, San Jose, CA) [30].

### 2.4. Cellular Thiol Levels Analysis

Cellular thiol levels were measured utilizing 5‐Chloromethylfluorescein diacetate (Green CMFDA) and flow cytometry analysis. Cells were washed with PBS and incubated at 37°C for 15 min with 4 µM CMFDA in FCS‐free DMEM. After incubation, the CMFDA medium was replaced with fresh FCS‐free DMEM. Thirty minutes later, the cells were trypsinized, centrifuged at 685 × *g* for 5 min at 4°C, and resuspended in PBS. Cells were analyzed by a flow cytometer [31].

### 2.5. Lipid Peroxidation Analysis

Lipid peroxidation was measured using a TBARS (thiobarbituric acid reactive substances) assay and HPLC‐fluorescent analysis. AML12 cells were trypsinized, washed with PBS, and centrifuged at 5000 rpm for 5 min at 4°C. The pellet was dissolved in 500 µL of 12% TCA (trichloroacetic acid) and vortexed. After centrifugation at 3000 × *g* for 5 min at 4°C, 400 µL of the supernatant was transferred to a new Eppendorf tube, and the pellet was stored at −20°C for protein quantification. For the TBARS assay, 250 µL of 20 mM TBA (thiobarbituric acid) in 50% acetic acid was added to the supernatant. The samples were incubated at 100°C for 60 min. 30 *μ* L of filtered samples were injected and separated with a C‐18 Phenomenex column, model RP‐18, and detected with an HPLC (Merck/Hitachi HPLC system, LaChrom L 7100 pump, and LaChrom Fluorescence Varian ProStar 363 detector) set at 532 nm excitation and 553 nm emission. The mobile phase consisted of a 35:65 (*v*/*v*) mixture of methanol and 0.05 M potassium phosphate buffer, pH 7, and the flow rate was 0.6 mL min^−1^. Malondialdehyde (MDA) standard solutions (produced by tetraethoxypropane) were used to generate a standard curve. For protein quantification, the remaining pellet was treated with 130 µL of 0.3 M KOH, heated at 65°C for 30 min, and then analyzed for protein content utilizing the Bradford assay.

### 2.6. Fatty Acid Profile: GC‐FID Analysis of Fatty Acids Methyl Esters (FAMEs)

The cells collected following trypsinization were washed with PBS and centrifugation at 1500 rpm for 4 min (Thermo Scientific, Heraeus Megafuge 16R, Osterode am Harz, Germany) at 4°C. The PBS was aspirated, and the samples were frozen overnight at −20°C. Lipids were converted into FAMEs by trans‐methylation in the media of methanol/HCl (1%) with the addition of C17:0 as an internal standard. FAMEs were extracted from the reaction mixture with hexane. The GC–FID analysis of FAMEs was carried out using a gas chromatograph equipped with a flame ionization detector (model 7890A, Agilent). FAMEs were separated on a DB‐23 capillary column (60 m, 0.25 mm, 0.25 µm, Agilent); hydrogen was used as a carrier gas. The analysis was performed by the Interdepartmental Analytical Unit (TSABAM) at the Faculty of Agriculture, Food, and Environment, Rehovot.

### 2.7. Intracellular Lipid Levels

For intracellular lipids, Nile Red (purchased from Sigma) staining assays and flow cytometry analysis were used. AML12 cells were trypsinized, washed with PBS, centrifuged for 5 min at 1500 rpm at 4°C, and resuspended in 500 µL PBS. A 1 mg/mL stock solution of Nile Red in DMSO was prepared. Nile Red was diluted to 2 µg/mL in PBS, and 500 µL were added to each sample to achieve a final 1 µg/mL concentration. Samples were incubated at room temperature for 20 min in the dark, then centrifuged, washed with PBS, and resuspended in 0.5 mL PBS. Cells were filtered through a 90‐µm mesh grid and analyzed by flow cytometry. (Stratedigm, SE520EON, San Jose, CA). The analysis was performed with Cell CapTure Analysis software version 4.1 (Stratedigm, San Jose, CA).

### 2.8. Gene Expression

Total RNA was isolated using the Tri Reagent (Sigma–Aldrich, Rehovot, Israel) solution. One microgram of total RNA was converted into cDNA. Real‐time PCR was performed using the 7300 Real‐Time PCR system (Applied Biosystems, Singapore) and carried out with the PerfeCTa SYBR Green FastMix (Quanta Bio, Gaithersburg, MD, USA). 18S was used as the control gene for real‐time PCR. The sequences of the primers used appear in Table 1.

**Table 1:** Primer sequences

	Accession number	Reverse	Forward
18s	NR_003278.3	5’‐CCTCAGTTCCGAAAACCAAC‐3’	5’‐ACCGCAGCTAGGAATAATGG‐3’
HO1	NM_010442.2	5’ ‐CTTCCAGGGCCGTGTAGAT‐3’	5’‐CAGAAGGGTCAGGTGTCCA‐3’
GCLC	NM_010295.2	5’‐TCGCCTCCATTCAGTAACAA‐3’	5’‐CGAGGTGGAGTACATGTTGG‐3’
GSTA1	NM_008181.3	5’‐TGCAGCTTCACTGAATCTTGAAAG‐3’	5’‐CCCCTTTCCCTCTGCTGAAG‐3’
CYP7A1	NM_007824.3	5’‐ TTGTTCAAGACCGCACATAAAGCC‐3’	5’‐CGTAGACGGATCAGTTCAGAGACC‐3’

^∗^18s‐ 18s ribosomal RNA; HO1‐ Heme oxygenase 1; glutamate‐cysteine ligase catalytic subunit; GSTA1‐ glutathione S‐transferase alpha 1; CYP7A1‐ cholesterol‐7 *α*‐hydroxylase (cytochrome P450 7A1).

### 2.9. RNA‐Sequencing

Total RNA was extracted using the Tri Reagent solution (Sigma–Aldrich, Rehovot, Israel). RNA was sent to the Genomic Applications Laboratory, the Core Research Facility, the Faculty of Medicine, the Hebrew University of Jerusalem, Israel. RNA quality was determined by Agilent 2200 TapeStation analysis. For mRNA library preparation, a KAPA Stranded mRNA‐Seq Kit was used (Kapa Biosystems, KK8421) according to the manufacturer’s recommendations.

### 2.10. RNA‐Sequence Analysis

Raw reads were aligned to the mouse transcriptome and genome version GRCh39 with annotations from Ensembl [32] release 106 using TopHat v2.1.1 [33]. Counts per gene quantification were done using htseq‐count v2.01 [34]. Genes with counts below 10 across all samples were filtered out. Normalization and differential expression analysis were done with the DESeq2 package v1.36.0 [35]. Differentially expressed (DE) genes between E7KC and E samples were defined by applying a significance threshold of false discovery rate (FDR) corrected *p*‐value < 0.1.

### 2.11. SiRNA for CYP51

To downregulate the expression of the CYP51 gene, we used the TriFECTa kit DsiRNA protocol by Integrated DNA Technologies, Inc. (IDT). Each of the three predesigned DsiRNAs‐ mm.Ri.Cyp51.13.1(#:239213079), mm.Ri.Cyp51.13.2 (#:239213082), mm.Ri.Cyp51.13.3 (#:239213985) was used equimolarly at a final total concentration of 10 nM. To perform the transfection, we utilized the jetPRIME transfection reagent kit. The cells were incubated for 48–72 h post‐transfection prior to further procedures.

### 2.12. Western Blot Analysis CYP51

The protein content was measured using the BCA method. 100 µg of proteins were separated on 10% SDS‐polyacrylamide gel, subjected to electrophoresis, and transferred to nitrocellulose membranes. The membranes were blocked with 5% BSA in TBST (Tris‐buffered saline with Tween) at room temperature for 1 h. After blocking, the membranes were incubated overnight at 4°C with the primary antibodies: Anti‐CYP51A1/CYP51 (AB‐ab210792, 1:40,000) and Anti‐GAPDH (Cell Signaling, Danvers, Massachusetts, USA, 14C10, 1:80,000). Afterward, the membranes were washed three times for 15 min each with TBST. Secondary antibodies: peroxidase‐conjugated goat anti‐rabbit IgG (cat# 111‐035‐144) for CYP51 and GAPDH, all at a dilution of 1:5,000. The resulting band intensity was analyzed using the Image Lab Gel Pro system (version 6.0.1, Bio‐Rad). The CYP51 band intensities were normalized to the GAPDH band intensity.

### 2.13. Statistical Analysis

Values are presented as means ± SD. Data were analyzed using one‐way ANOVA followed by the Tukey–Kramer HSD post‐hoc test, performed with JMP Pro 17 software (SAS Institute, Cary, NC, USA). A significance level of *p*  < 0.05 was considered statistically significant for all analyses.

## 3. Results

### 3.1. 7KC Prevents Ferroptotic Cell Death

The toxic effect of 7KC on AML12 hepatocytes was evaluated. No effect on cell viability was found at low concentrations of 20 µM and less, whereas concentrations of > 30 µM significantly decreased cell viability (Figure 1A). Ferroptosis was induced by employing two ferroptosis agents: Erastin, an inhibitor of the cystine‐glutamate antiporter system Xc‐, or RSL3, an inhibitor of GPX4. As shown in Figure 1B, the addition of 7KC protected against cell death caused by Erastin. On the other hand, when RSL3 was used as a ferroptosis mediator (Figure 1C), 7KC’s protective effect against RSL3 was smaller. Results were further corroborated by live images of the PI staining of undetected cells, demonstrating a profound protective effect for 7KC under ferroptosis conditions (Figure S1). 7KC protected dose‐dependently against Erastin‐induced cell death (Figure 1D). The most effective protection under Erastin treatment was seen at 20 and 10 µM 7KC (Figure 1D). Toxic higher concentrations were detrimental to the cells in the presence of Erastin, and an additive effect of cell death was displayed (Figure 1D). We compared the effect of 7KC with that of 20 µM cholesterol. Cholesterol failed to mitigate cell death induced by Erastin after 24 h of treatment (Figure 1E). We further tested the effect of 7KC in another ferroptosis system, using high glutamate levels for 18 h, in HT4 neurons. For this, a low‐toxic concentration of 10 and 20 *μ* M of 7KC was utilized (Figure S2). 7KC at nontoxic concentrations significantly alleviated ferroptosis cell death induced by glutamate in the HT4 cells (Figure S2).

Figure 1The effect of 7KC on cell viability in ferroptosis hepatocytes. AML12 cells’ viability was evaluated using the propidium iodide (PI) exclusion method. (A) Viability of AML 12 hepatocytes treated with 10, 20, 30, 40, and 50 µM 7KC for 24 h. (B) Viability of AML 12 cells was treated with 20 µM Erastin and 20 µM 7KC for 6, 12, 18, and 24 h. (C) Viability of AML 12 hepatocytes treated with 5 µM RSL3 and 7KC 20 µM for 12 h. (D) Viability of AML 12 hepatocytes treated with 5, 10, 20, 50, and 100 µM 7KC with or without 20 µM Erastin for 24 h. (E) Viability of AML‐12 treated with 20 µM Erastin, 20 µM cholesterol, and 20 µM 7KC for 24 h. (10,000 cells were counted). *n* = 4, means with different letters are statistically different (*p*  < 0.05).

**Figure:**
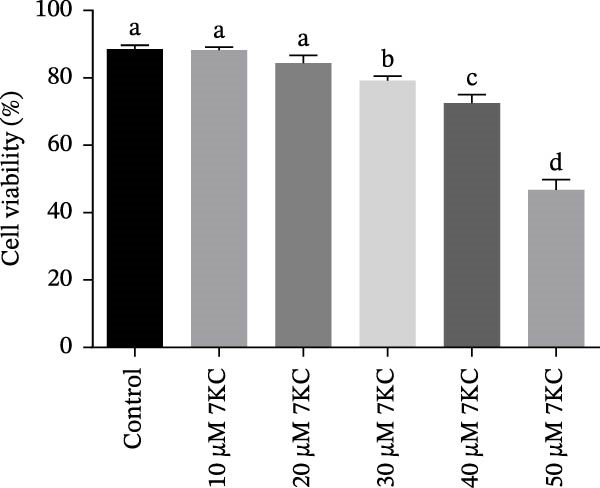
(A)

**Figure:**
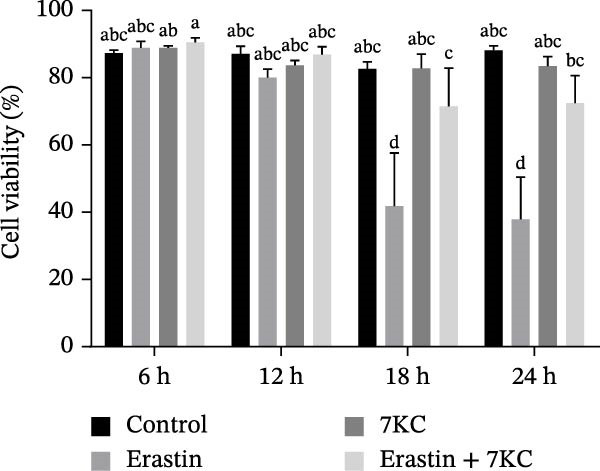
(B)

**Figure:**
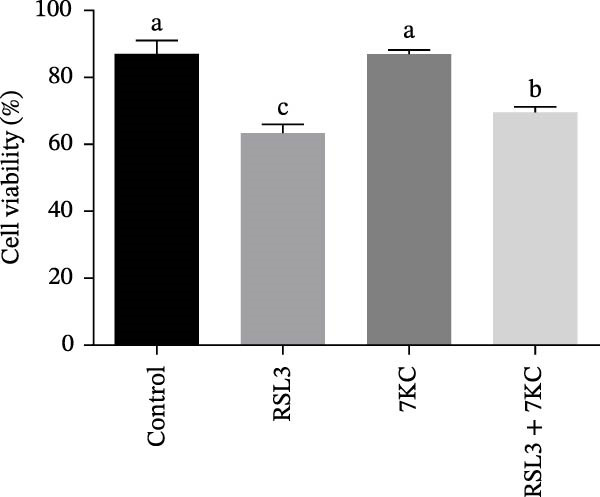
(C)

**Figure:**
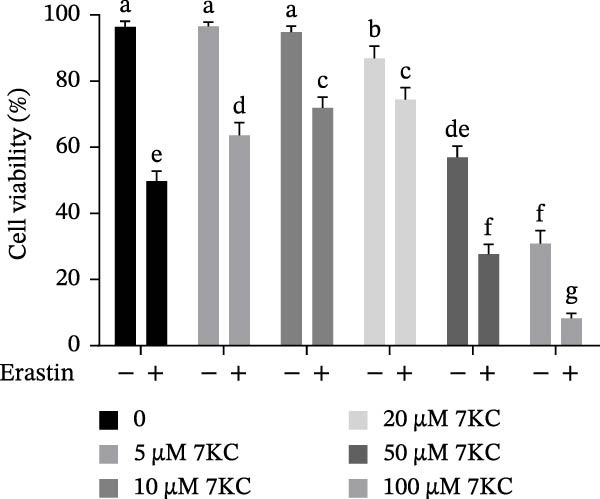
(D)

**Figure:**
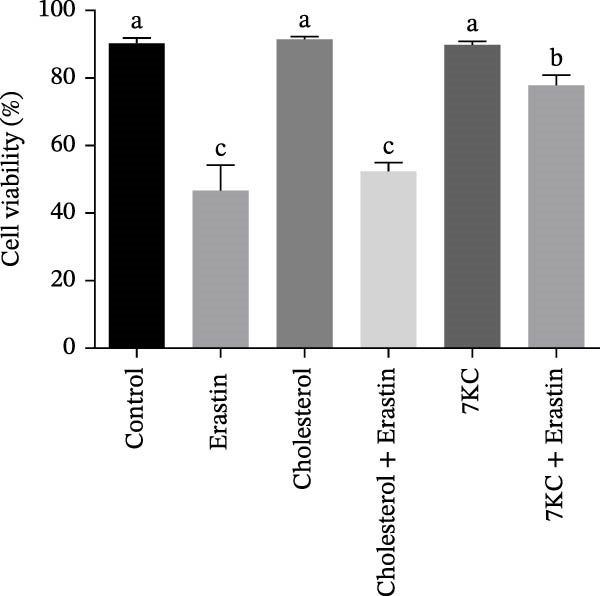
(E)

### 3.2. 7KC Restores Cellular Thiol Levels and Decreases Lipid Peroxidation During Ferroptosis in Hepatocytes

Erastin is known to inhibit system Xc− and cystine uptake into cells. Therefore, the influence of 7KC on cellular thiol levels under ferroptosis induction was evaluated. Erastin treatment alone for 12 h significantly decreased cellular thiol levels. Nevertheless, treatment of AML‐12 cells with 20 µM Erastin combined with 20 µM 7KC increased thiol levels by 1.3‐fold (Figure 2A). MDA is a marker of lipid peroxidation of polyunsaturated fatty acids (PUFAs) and serves as a biomarker for oxidative stress [36]. While treatment with Erastin increased MDA levels, the addition of 7KC to Erastin‐treated cells prevented the accumulation of MDA in these cells (Figure 2B). We also employed FAMEs GC analysis to characterize the effect of 7KC on cellular lipid profile. Following 6 h of ferroptosis induction, saturated fatty acids (SFAs) levels of pentadecanoic acid (C15:0) were elevated more by Erastin alone than by a combination of Erastin and 7KC. Palmitic acid (C16:0 PA) levels were increased by Erastin and to a greater extent by the combination of Erastin and 7KC (Figure 2C). Levels of the monounsaturated fatty acids (MUFAs) palmitoleic acid (C16:1 cis‐9), elaidic acid (C18:1 trans‐9), and vaccenic acid (C18:1 cis‐11) were significantly elevated with Erastin. However, only increased elaidic acid (C18:1 trans‐9) levels were found in the 7KC treatment group. (Figure 2D). An effect of 7KC was observed on PUFA levels. For the PUFAs linolelaidic acid (C18:2 trans‐9,12), arachidonic acid (C20:4), eicosapentaenoic acid (C20:5 cis‐5,8,11,14,17 EPA), and docosahexaenoic acid (C22:6 cis‐4,7,10,13,16,19 DHA), Erastin‐treated cells showed significantly lower levels compared with the combination of Erastin and 7KC as well as with the other treatments. 7KC augmented linoleic acid (C18:2 cis‐9,12) levels with no observed effect for Erastin (Figure 2E).

Figure 2The effect of 7KC on thiol levels and lipid peroxidation during ferroptosis in AML12 hepatocytes. (A) Thiols were measured with green CMFDA by FACS. AML12 hepatocytes were treated with 20 µM Erastin and 20 µM 7KC for 6 h (10,000 cells, *n* = 4). (B) MDA levels were measured in AML12 treated as described in materials and methods for 12 h (*n* = 4). (C–E) GC analysis of fatty acid methyl esters (FAMEs) was conducted to determine the fatty composition in AML‐12 cells. Cells were treated as described in material and methods for 6 h. Results are presented as micrograms of fatty acid per milligram of sample. (C) Saturated fatty acids (SFAs), (D) monounsaturated fatty acids (MUFAs), (E) polyunsaturated fatty acids (PUFAs) (*N* = 4). Means with different letters are statistically different (*p*  < 0.05).

**Figure:**
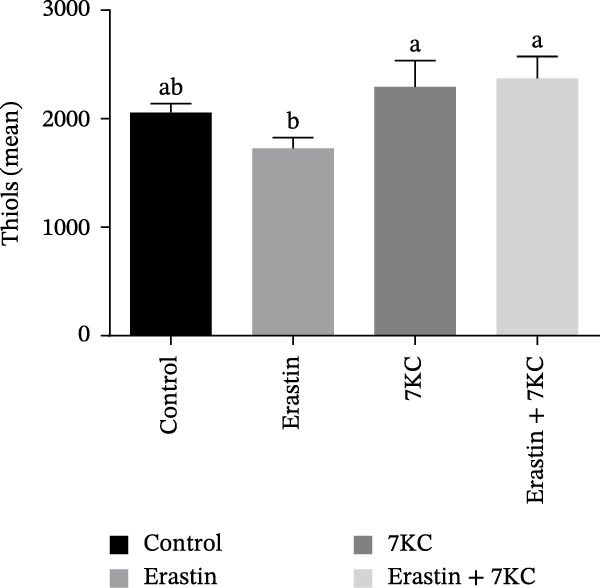
(A)

**Figure:**
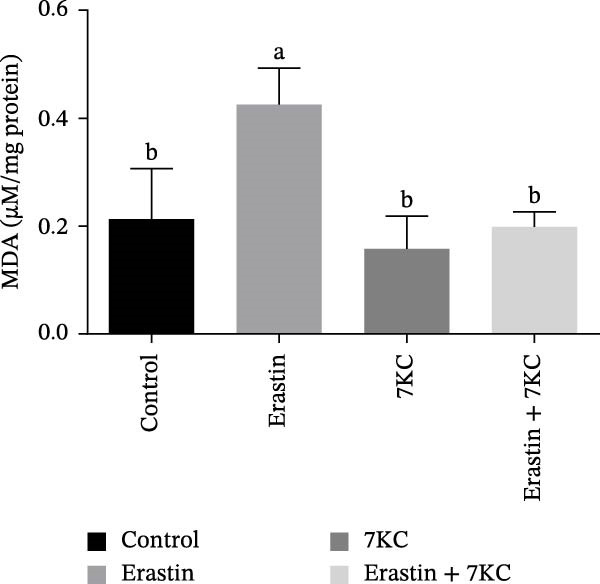
(B)

**Figure:**
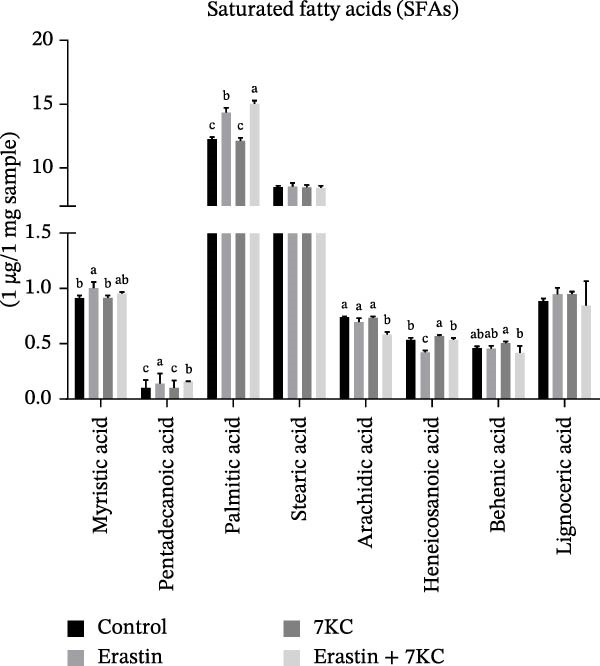
(C)

**Figure:**
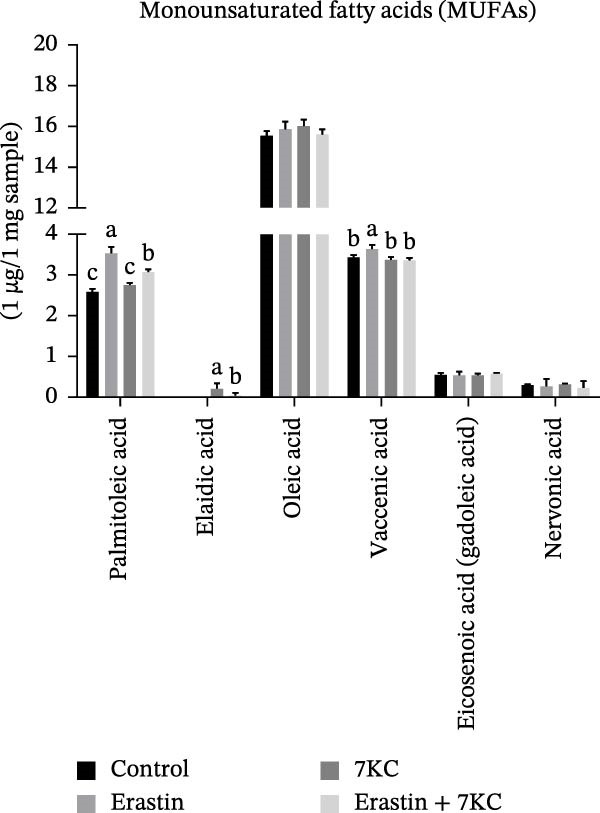
(D)

**Figure:**
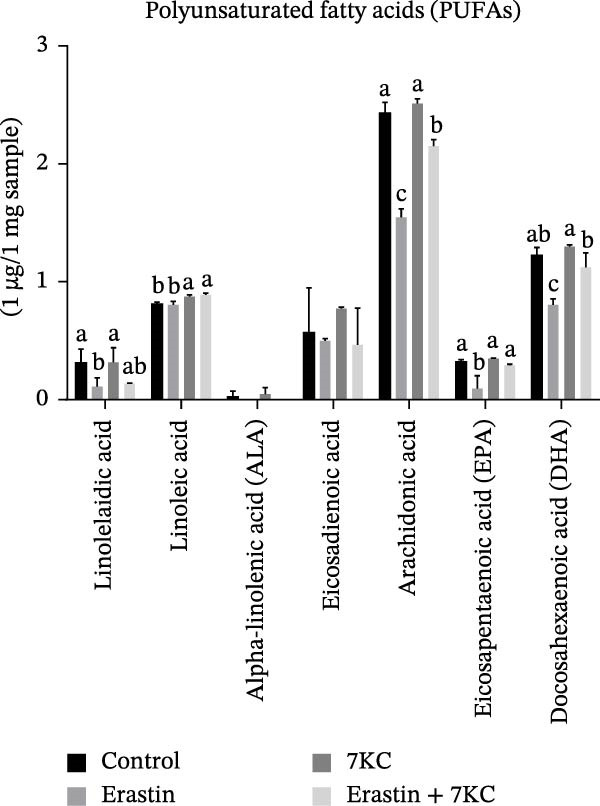
(E)

### 3.3. 7KC Differentially Affects NRF2‐Related Gene Expression During Ferroptosis in Hepatocytes

We investigated the ability of 7KC to up‐regulate the *NRF2* gene set, as this transcription factor is critically involved in the protective antioxidant response and is a vital ferroptosis regulator. The expression of gamma‐glutamylcysteine ligase catalytic (GCLC) unit, the rate‐limiting enzyme in glutathione synthesis, was increased in response to 7KC treatment under ferroptotic conditions compared to Erastin alone, thus indicating the hormetic effect of 7KC on this enzyme (Figure 3A). In contrast, the expression of glutathione S‐transferase *α*1 (GSTA1) and heme oxygenase 1 (HO1) was equally induced by Erastin and the 7KC combined with Erastin after 6 hours of exposure (Figure 3B,C). Given these results, the involvement of NRF2 was further examined using its novel specific inhibitor, ML385. As shown in Figure 3D, cell viability was similar whether NRF2 was inhibited or not, thus suggesting NRF2 is unlikely to mediate 7KC’s beneficial effect.

Figure 3The effect of 7‐ketocholesterol on NRF2 activation during ferroptosis in hepatocytes. NRF2‐related genes expression (A–C) in hepatocytes treated with 20 µM Erastin and/or 20 µM 7KC for 6 h. (*N* = 4). (D) Cell viability in hepatocytes treated with 20 µM Erastin and/or 20 µM 7KC with or without the NRF2‐specific inhibitor, ML385, for 6 h. Means with different letters are statistically different (*p* < 0.05).

**Figure:**
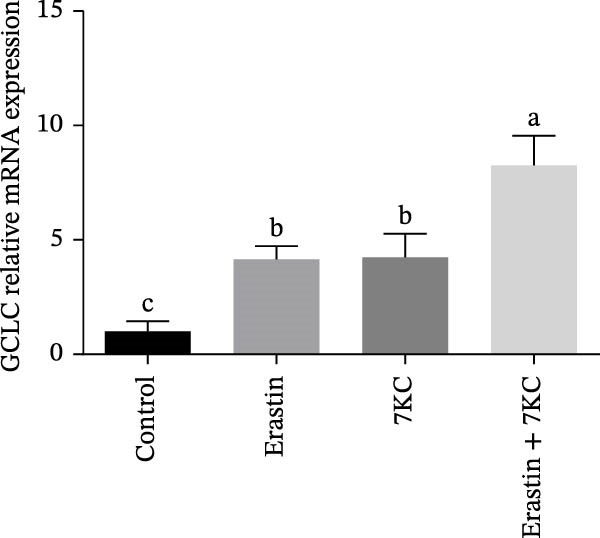
(A)

**Figure:**
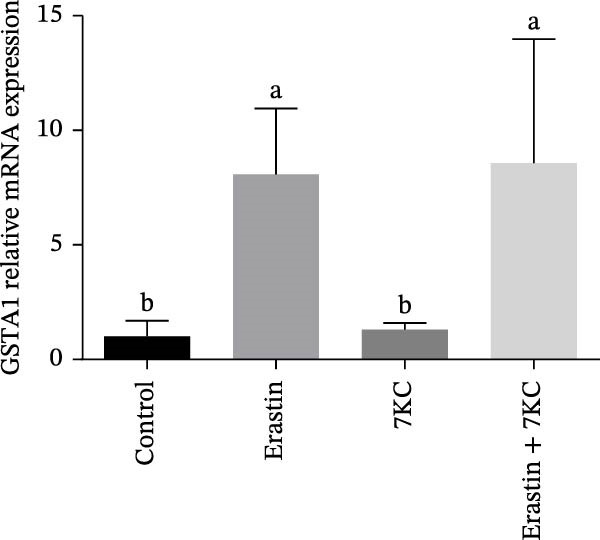
(B)

**Figure:**
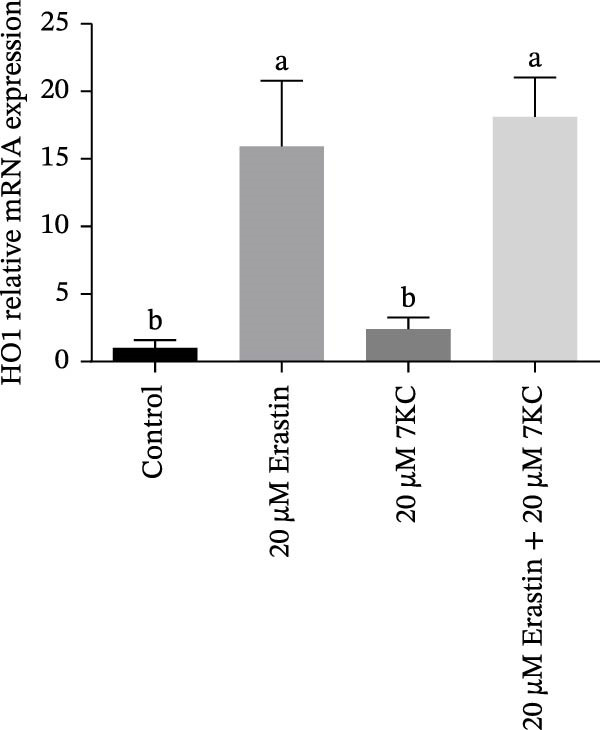
(C)

**Figure:**
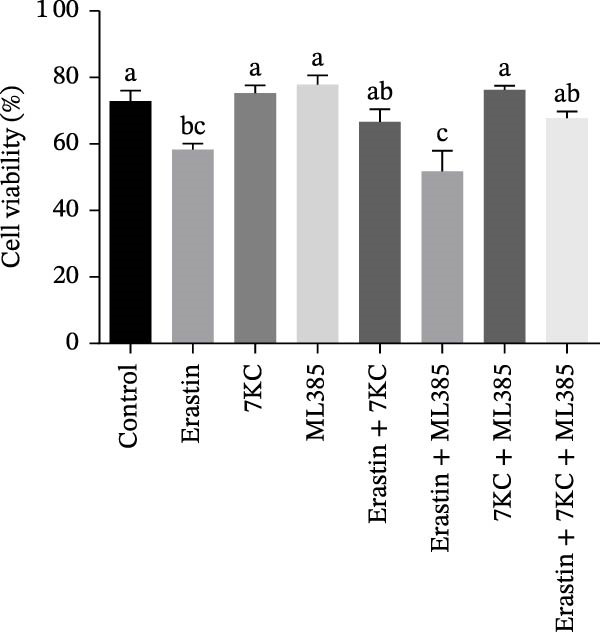
(D)

### 3.4. 7KC Decreased Intracellular Neutral Lipids and Cholesterol Metabolism During Ferroptosis in Hepatocyte Cells

The effect of 7KC on intracellular neutral and polar and neutral lipid accumulation under ferroptosis was assessed by Nile Red staining. Treatment with Erastin for 18 h significantly promoted steatosis, while the addition of 7KC to Erastin decreased this accumulation (Figure 4A,B). Previous studies have demonstrated the inhibitory effect of 7KC on HMG‐CoA reductase [25, 29] Therefore, we examined whether the abovementioned protective effect of 7KC is due to the inhibition of the cholesterol biosynthesis pathway. To address this, we compared the effects of 7KC to those of statins [37]. However, while 7KC protected the cells from Erastin, the combination of Erastin and statins further decreased cell viability compared to Erastin alone (Figure 4C), which indicates that inhibition of cholesterol synthesis did not protect hepatocytes from ferroptosis.

Figure 4The effect of 7KC on intracellular l lipids and cholesterol metabolism during ferroptosis in hepatocytes. Intracellular lipid levels were evaluated using Nile red staining and flow cytometry analysis (A) FL2‐neutral lipids and (B) FL3‐polar lipids. AML 12 hepatocytes were treated with 20 µM Erastin and 20 µM 7KC for 18 h. (C) Effect of statins on ferroptosis: AML12 cells were evaluated using the PI method. Cells were treated with 20 µM Erastin, 20 µM 7KC, and 20 µM statins for 24 h. Means with different letters are statistically different (*p*  < 0.05). (D) PCA plot of gene expression profiles under the treatments, for 6 h. Colors: red (E‐Erastin), blue (C‐control), yellow (7KC), and green (E7KC‐ Erastin + 7KC). PC1 and PC2 explain 53% and 25% variance, respectively. (E) AML12 hepatocytes were treated for 6 h. Heatmap showing expression levels of genes differentially expressed between E7KC and E samples. Red indicates higher, blue indicates lower expression. Hierarchical clustering by treatment. Significant genes were identified using DESeq2 with a *p*‐value < 0.1. (F,G) Normalized read counts representing gene expression profiles from RNA‐seq analysis. Differential expression analysis was performed using DESeq2, with significance thresholds indicated as  ^∗^ *p* ≤ 0.05,  ^∗∗^ *p* ≤ 0.01,  ^∗∗∗^ *p* ≤ 0.001.

**Figure:**
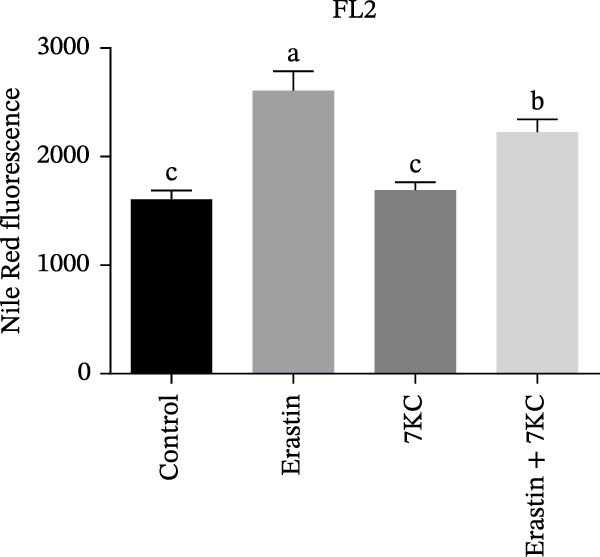
(A)

**Figure:**
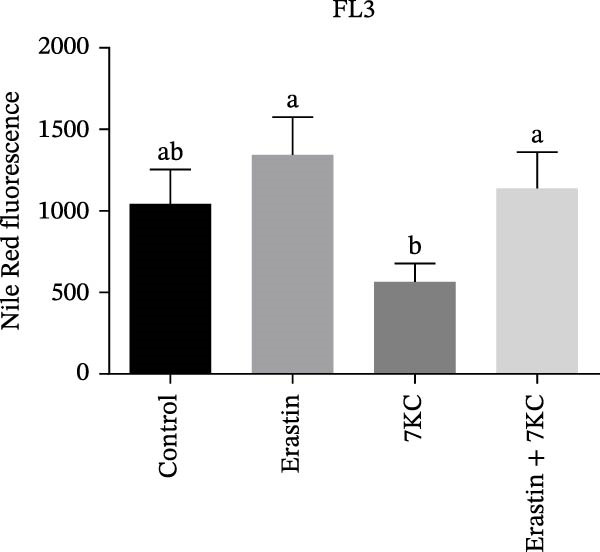
(B)

**Figure:**
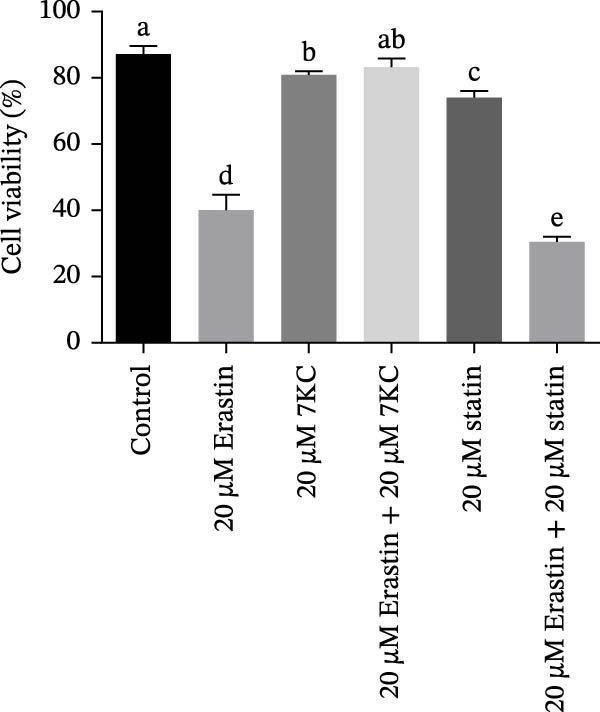
(C)

**Figure:**
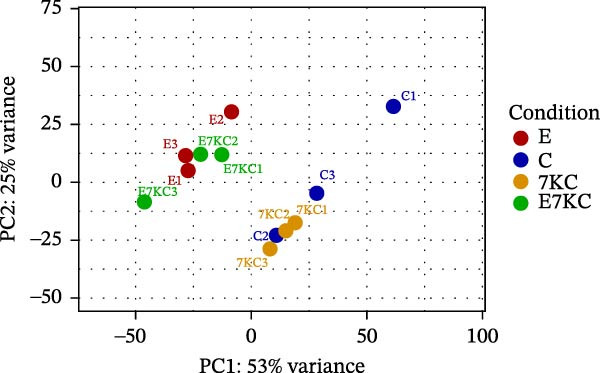
(D)

**Figure:**
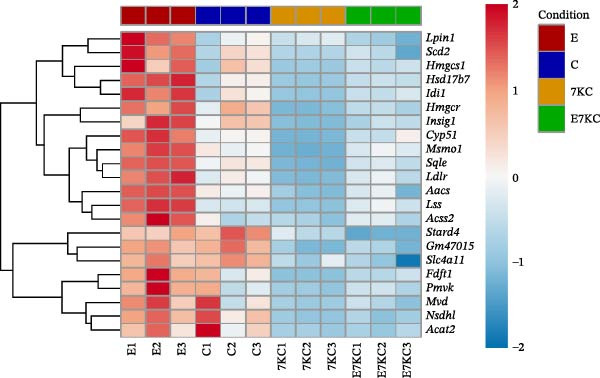
(E)

**Figure:**
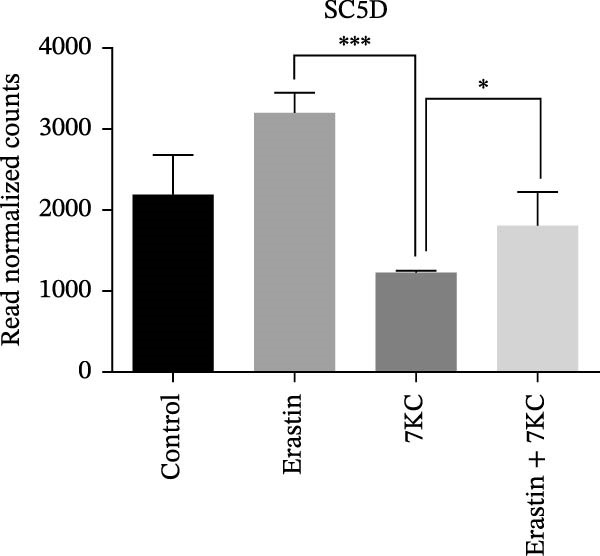
(F)

**Figure:**
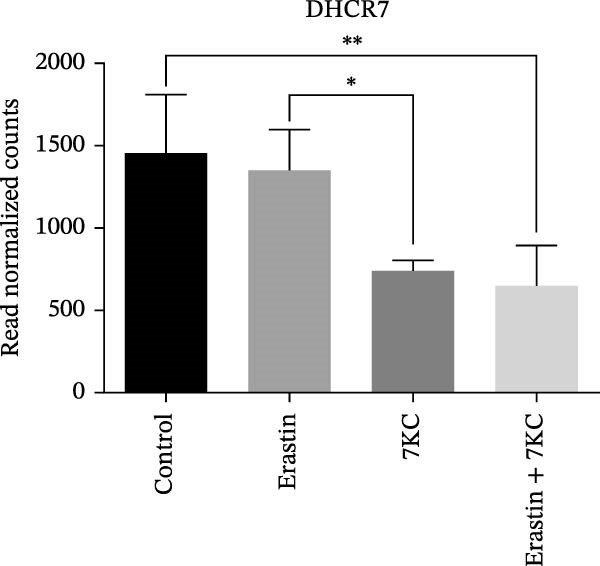
(G)

To achieve a better understanding of the mechanisms of action of 7KC, gene expression by RNA sequencing (RNA‐seq) in cells that were treated with Erastin and/or 7KC for 6 h was performed. Principal component analysis (PCA) demonstrated that PC1 explained 53% of the variance, while PC2 explained 25%. The control samples clustered similarly to those of the 7KC samples, suggesting a comparable metabolic state. Conversely, the Erastin treatment samples clustered similarly to that of the Erastin with 7KC treatment samples (at nontoxic concentration), which might imply a similar response to these treatments (Figure 4D). Nevertheless, despite the apparent resemblance between the Erastin treatment and the Erastin and 7KC combination, a group of 22 genes was found to be DE (Table S1). The combination of Erastin and 7KC markedly downregulated cholesterol synthesis genes such as *3-Hydroxy-3-Methylglutaryl-CoA Synthase 1 (Hmgcs1)*, which catalyzes the condensation of acetyl‐CoA with acetoacetyl‐CoA to form HMG‐CoA, 3‐hydroxy‐3‐methylglutaryl‐CoA reductase (Hmgcr), which catalyzes the conversion of HMG‐CoA to MVA [27]. In contrast, Erastin treatment alone upregulated these genes (Figure 4E). In the terminal stages of cholesterol synthesis, Erastin was found to upregulate the expression of sterol C5‐desaturase (SC5D), the enzyme catalyzing the conversion of lanosterol to 7DHC. In contrast, treatment with 7KC reduced SC5D expression relative to the combination treatment with 7KC and Erastin (Figure 4F). The expression of 7DHCR, the enzyme responsible for the conversion of 7DHC to cholesterol, was decreased following 7KC treatment with and without Erastin compared to control or to Erastin pro‐ferroptotic treatment (Figure 4G).

### 3.5. The Inhibition of CYP51 Did Not Protect Against Ferroptosis

We further evaluated whether inhibition at the upper stages of the cholesterol synthesis pathway has any effect. Lanosterol 14‐alpha demethylase (cytochrome P450(51), CYP51A1) catalyzes one of the key steps in cholesterol biosynthesis [38] and is among the genes that were profoundly affected by the treatments. Since the expression of CYP51 was hindered by 7KC, we aimed to determine whether inhibiting CYP51, using the siRNA, would protect cells from ferroptosis. CYP51 protein levels were upregulated by Erastin treatment for 24 h but not after 6 h of treatment. The addition of 7KC to Erastin moderated this induction, although it did not affect the CYP51 protein level at an earlier time of 6 h. As expected, groups treated with siRNA against CYP51 exhibited much lower levels of this protein, with only a minor effect for Erastin treatment in these cells (Figure 5A,B). As shown in Figure 5C, downregulation of CYP51 did not protect the cells from ferroptosis, as there was no significant difference between the effects of Erastin alone and of CYP51 siRNA treatment with Erastin. Additionally, no difference was observed between the combination of Erastin and 7KC and the combination with siRNA.

Figure 5The effect of 7KC and CYP51 siRNA in ferroptosis: CYP51 protein accumulation in AML12 hepatocytes treated with 20 µM Erastin, 20 µM 7KC for 6 h. (A) Transfection with anti‐CYP51 siRNA for 48 h. (B) Transfection with anti‐SYP51 siRNA for 72 h. (C) AML12 cells viability was evaluated using the PI method. Cells were treated with anti‐CYP51 siRNA for 48 h, and after 24 h with 20 µM Erastin and 20 µM 7KC for an additional 24 h. (*n* = 4) Means with different letters are statistically different (*p*  < 0.05).

**Figure:**
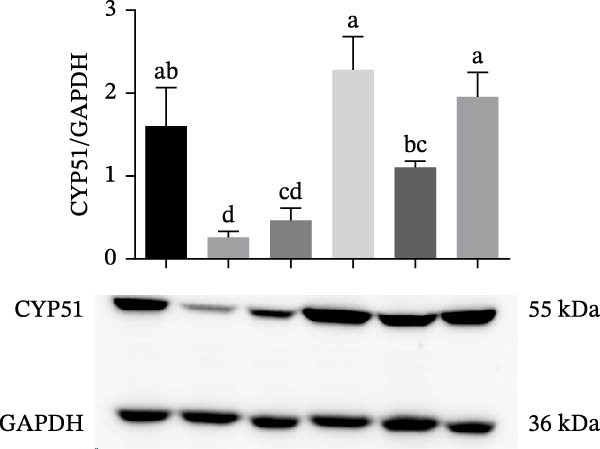
(A)

**Figure:**
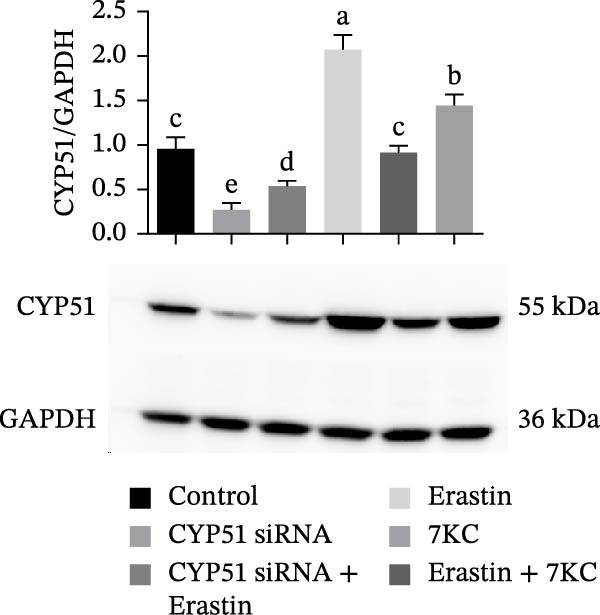
(B)

**Figure:**
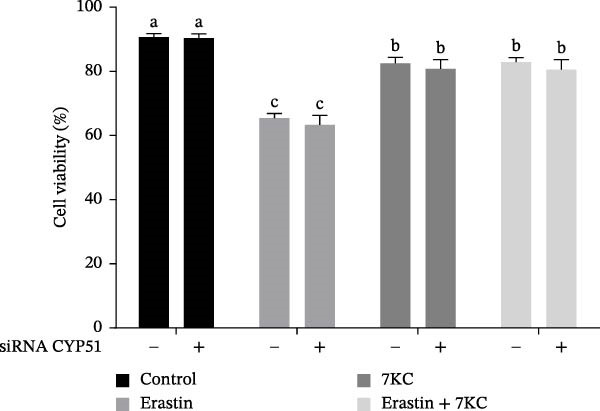
(C)

### 3.6. 7KC Demonstrated a Better Protective Effect Than 7DHC During Ferroptosis in Hepatocytes

Several studies have shown that 7DHC, an intermediate metabolite in cholesterol biosynthesis, protects against ferroptosis [39, 40]. 7DHC can be metabolized to 7KC by the enzyme CYP7A1 [7]. To differentiate the effects of 7DHC from those of 7KC, we also utilized CdCA to decrease the expression of CYP7A1 [41] and, accordingly, prevent the conversion of 7DHC to 7KC. As shown in Figure 6A, CdCA decreases the expression of CYP7A1. The addition of 7KC ameliorated cell death after 24 h of ferroptosis induction by Erastin (Figure 6B), whereas the addition of 7DHC did not improve cell survival under the same conditions [41]. Moreover, the addition of 7DHC while simultaneously inhibiting its oxidation toward 7KC failed to affect cell viability. Interestingly, the combination of 7KC and CdCA improved cell survival even more than 7KC alone under ferroptosis conditions, which indicates that controlling low doses of 7KC is better for protecting cells (Figure 6B, Figure S3).

Figure 6The effect of 7KC and 7DHC in ferroptosis. (A) *CYP7A1* gene expression in hepatocytes treated with 20 µM 7DHC, 20 µM 7KC, and 100 µM CdAC for 24 h (*n* = 4). (B) Cells were evaluated using the propidium iodide (PI) method. AML 12 hepatocytes were treated with 20 µM Erastin, 20 µM 7KC, 20 µM 7DHC, and 100 µM CdAC for 24 h. (10,000 cells, *n* = 4). Means with different letters are statistically different (*p*  < 0.05).

**Figure:**
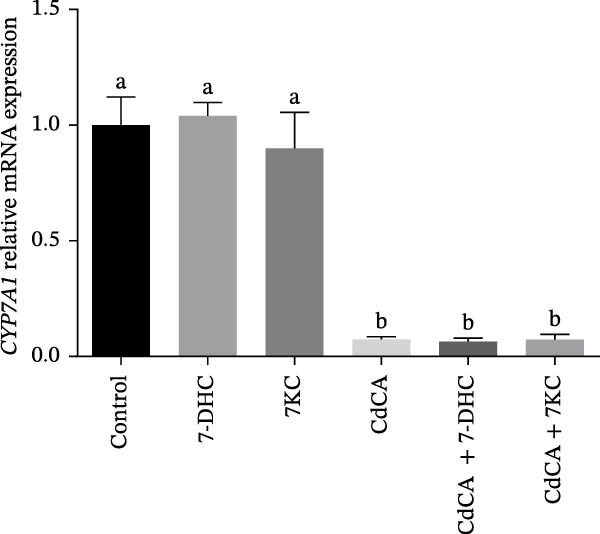
(A)

**Figure:**
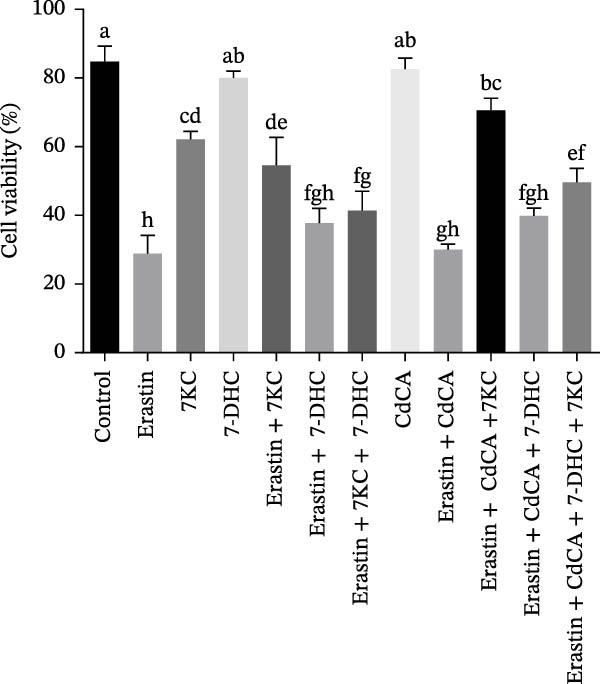
(B)

## 4. Discussion

This study is the first to demonstrate the impact of subtoxic levels of 7KC on ferroptosis. While mostly known as a toxic, pro‐apoptotic with no known biological function oxysterol, we found that treatment with 7KC, but not cholesterol, at low concentrations, protects against cell death induced by ferroptosis agents, such as Erastin or glutamate. In total 20 µM of 7KC was used in our model to achieve maximum protection. Although 20 µM of 7KC is considered physiologically high, 7KC is less toxic in hepatocytes [42], and concentrations around 50 and 100 µM are needed for toxic effect. Interestingly, at toxic concentrations, additive killing effect was observed in the presence of Erasin, which is in line with the concept of hormesis. Additionally, we observed that cell death stress by thiol limitation induced by Erastin was an inducer of neutral lipid accumulation and steatosis. The hormetic, favorable effect of 7KC was found to be associated with the improvement of intracellular thiol levels and the restriction of stress‐induced hepatic steatosis and cholesterol synthesis. This is a unique finding given that this molecule is without antioxidant properties by itself or chelation capacity but, nevertheless, can ameliorate ferroptotic cell death at low concentrations. Unlike chain‐breaking antioxidants, 7KC is not a direct free radical scavenger and cannot inhibit lipid peroxidation progression directly. This might be elucidated by its inefficiency in blocking the oxidative stress induced by RSL3 as a GPx4 inhibitor [43]. Conversely, the hormetic effect against Erastin appears to be a consequence of its ability to stimulate thiol production by GCLC and subsequently protect against thiol depletion.

Increased intracellular lipid accumulation following exposure to a pro‐ferroptosis agent was evident by Nile red staining and gene expression of cholesterol synthesis genes, thus suggesting an elevation in cholesterol levels during ferroptosis. The addition of 7KC significantly lowered hepatocyte steatosis and cholesterol gene expression. However, this lipid‐inhibitory effect of 7KC appears to be dissociated from the mechanism by which 7KC exerts its protective effect under ferroptosis. Indeed, inhibition of the MVA pathway by statins, or by siRNA for CYP51, did not protect against ferroptosis but rather made the cells more susceptible to cell death. The ability of 7KC to protect against ferroptosis despite its negative effect on cholesterol synthesis may suggest that low levels of this compound relinquish the need to accumulate lipids or to alter their metabolic flux as a cellular protective response against ferroptosis. Adding cholesterol and changing the metabolic flux of the MVA pathway may protect cells against ferroptosis by increasing both CoQ10 and squalene levels [40]. However, at 20 µM, cholesterol was ineffective as a ferroptosis inhibitor compared to the significant effect of 7KC. The lack of ability of 20 µM cholesterol to attenuate cell death may indicate no change in the metabolic flux of the cell by cholesterol at the levels being used.

Several lines of evidence have highlighted the importance of 7DHC, an intermediate metabolite in cholesterol biosynthesis through the Kandutsch–Russell pathway, as a protective metabolite against ferroptotic cell death [39, 40]. 7DHC is produced by SC5D and further metabolized by 7DHC reductase to form cholesterol. It was suggested that 7DHC regulates ferroptosis by using its conjugated diene structure to hamper phospholipid autoxidation, and thus effectively supports the maintenance of functional plasma and mitochondrial membranes [39]. Here, we demonstrate that 7KC, a side metabolite of 7DHC by the activity of CYP7A1, has a more advantageous protective impact under a pro‐ferroptosis setting compared to its precursor, though concentrations should be kept under close restriction to avoid toxicity. The hormesis effect of 7KC needed for protection is very delicate since exogenous levels, along with internal production of 7KC from 7DHC, were less protective against Erastin compared to when internal production was held by the CYP7A1 inhibitor and natural FXR agonist, chenodeoxycholic acid. Importantly, it should be stressed that 7KC can be generated via other mechanisms, not requiring 7DHC or CYP7A1. 7KC is also the product of another enzymatic pathway, catalyzed by 11*β*‐HSD2, which is physiologically relevant, as well as nonenzymatically under oxidative stress [7]. Our result cannot exclude the significance of these paths in 7KC generation or source in the current setting. Supporting this notion, inhibition of CYP7A1 did not exacerbate cell injury during Erastin‐induced ferroptosis. Thus, it can be inferred that 7KC was still continuously generated from an alternative source that bypassed the necessity of CYP7A1 and served to compensate for its shortage. Further studies are needed to elucidate the exact role of other mechanisms as a source of 7KC during ferroptosis.

**Scheme 1:**
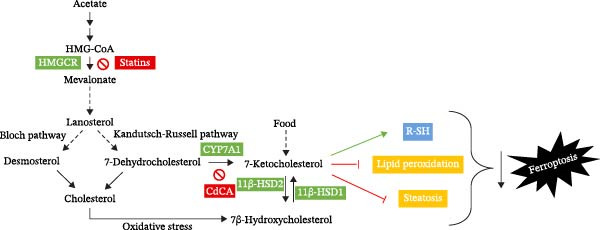
Protective mechanism of 7KC at low, nontoxic concentrations against ferroptosis.

The defensive effect of 7KC against Erastin‐induced ferroptotic cell death was associated with the normalization of cellular thiol levels, which corresponded with lower lipid peroxidation as revealed by MDA levels (Scheme 1). While the precise mechanism is not clear, enhanced cellular thiol levels probably mediate the alleviating effect of 7KC on cell death. Importantly, the positive impact of 7KC on thiol and lipid peroxidation appears to arise from low and not high concentrations of this compound, as elevated, toxic levels of 100 µM were found to lower GSH [42]. The restoration of cellular thiol status by 7KC was associated with an augmented upregulation of GCLC expression, but not other *NRF2/NRF1*‐dependent genes that were similarly affected by Erastin with or without 7KC. These findings indicate that the protective effects of 7KC during ferroptosis do not require NRF2 activation. Should GCLC indeed participate in the beneficial effect of 7KC, its expression seems to be regulated in an NRF2‐independent mechanism [44]. More work is needed to elucidate the precise mechanism by which 7KC mediates its effect on cellular thiol.

In light of the discrepancy in lipid peroxidation and in an attempt to further characterize the effect of 7KC in our setting, the fatty acid profile was evaluated. Treatment with Erastin led to a decrease in several key PUFAs, including arachidonic acid, EPA, and DHA, while these fatty acid levels were un‐ or only modestly affected in the copresence of 7KC. These fatty acids contain several double bonds and, as such, are more susceptible to oxidant attack. Thus, the observed differences between Erastin alone and Erastin with 7KC may be the consequence of more PUFAs peroxidation in the former, given the augmented oxidative environment in this setting. Yet, enzymatic involvement in PUFAs peroxidation in our setting cannot be ruled out. Lipoxygenase (LOX) enzymes have been implicated in the promotion of ferroptosis [45]. Although exposure to high (30 µM) 7KC concentration was shown to enhance LOX and cyclooxygenase expression [46], the outcome of low 7KC concentrations in the context of ferroptosis on these oxygenases is unknown. Regardless of the cause, specific peroxidation of the abovementioned fatty acids in the cellular membrane has been implicated in the induction or enhancement of ferroptosis [47]. Interestingly, the notion of lesser membrane PUFAs peroxidation during ferroptosis in the presence of 7KC corresponds with reduced lipid accumulation in this group. Expanded incorporation of PUFAs in membrane phospholipids promotes lipid peroxidation and further supports ferroptosis. This response can lead to lipid accumulation as cells produce more lipids to repair membrane damage and maintain stability under stress [48]. In the context of our findings, the additional presence of 7KC appears to counteract this lipid buildup under Erastin treatment due to lower membrane peroxidation in this group.

With the continuation of the essence of fatty acid saturation, previously published works conducted in cancer cells demonstrated that a high MUFA/PUFA ratio elicits beneficial effects against ferroptosis [49–51]. The influence of 7KC on the overall MUFA/PUFA ratio cannot be ascertained here. Nevertheless, this mechanism does not appear to mediate the 7KC protective effect in the current model, considering that key desaturase expression was not markedly altered. In fact, stearoyl‐CoA desaturase‐2 (SCD2), a less dominant hepatic isoform than SCD1, which catalyzes the desaturation of stearic and palmitic FA and hence gives rise to MUFA levels [49–51], was actually decreased rather than increased.

In conclusion, this study identifies a new role for 7KC as a potential protector against ferroptosis at low hormetic concentrations. Subtoxic levels of 7KC ameliorated cellular thiol levels, an effect associated with elevated GCLC expression, reduced lipid peroxidation, and lower steatosis (Scheme 1). Protection appears to be derived from 7KC rather than its precursor 7DHC. Further studies are necessary to confirm this hormetic mechanism of action, as well as to adequately characterize the specific contribution of each of the other differently expressed genes in the observed effect. Advancing our understanding of the involvement of oxysterols in liver cell death may offer new therapeutic possibilities for oxysterols.

## 5. Future Perspective

As 7KC dose‐dependently prevents ferroptosis at low concentrations, it could be utilized as a therapeutic agent. However, even at low concentrations, and depending on the age and the comorbidities in the patients, 7KC can accumulate or be degraded. Therefore, there is an interest in identifying molecules with improved therapeutic windows with the backbone of 7KC (7KC derivatives and metabolites) [7], which could be as efficient or more efficient than 7KC at low and very low concentrations and which have lower toxic effects at elevated concentrations [52, 53].

## Author Contributions

Oren Tirosh and Sarit Anavi are responsible for oversight and leadership responsibility for the research activity planning and execution, including mentorship external to the core team. Zecharia Madar is responsible for the acquisition of the financial support for the project leading to this publication. Oren Tirosh, Sarit Anavi, and Zecharia Madar are responsible for the ideas, formulation, or evolution of overarching research goals and aims. Nicole Giltman and Haim Zeigerman are responsible for the preparation, creation, and/or presentation of the published work, specifically writing the initial draft (including substantive translation). Nicole Giltman, Haim Zeigerman, and Oren Tirosh are responsible for the development or design of methodology and the creation of models.

## Funding

No specific funding was received for this manuscript.

## Disclosure

No persons or third‐party services were involved in the research or manuscript preparation who are not listed as an author and have not been acknowledged. An abstract of the current study was presented as a poster at the EASL SLD Summit, 23‐25. https://easl.eu/wp-content/uploads/2025/01/EASL_SLD_Summit_2025-Abstract-book-FINAL.pdf

## Conflicts of Interest

The authors declare no conflicts of interest.

## Supporting Information

Additional supporting information can be found online in the Supporting Information section.

## Supporting information


**Supporting Information** The supporting section describes additional analyses of 7KC effects on ferroptosis and lipid metabolism: Figure S1 shows the effect of 7KC on cell viability in ferroptotic hepatocytes, where AML12 cells were treated with 7KC and erastin for 24 h and viability was assessed by propidium iodide (PI) exclusion, with red‐stained cells indicating dead or damaged cells and unstained cells indicating viable cells; Figure S2 presents the effect of 7KC on cell viability in glutamate‐induced ferroptosis of HT4 neuronal cells, assessed by PI staining, including (A) dose‐dependent treatment with 7KC (10–50 µM) and (B) treatment with 10 mM glutamate in the presence of 10 or 20 µM 7KC for 18 h, with 10,000 cells counted per condition (*n* = 4) and statistically significant differences denoted by different letters (*p* < 0.05); Table S1 lists of differently affected genes by 7KC and erastin predominantly involved in cholesterol, lipid, and sterol metabolism, including key enzymes of the mevalonate pathway (Hmgcs1, Hmgcr), downstream isoprenoid‐processing enzymes (Mvd, Pmvk, Idi1), acetyl‐CoA–providing enzymes (Aacs, Acss2), sterol biosynthesis enzymes (Fdft1, Sqle, Lss, Nsdhl, Msmo1, Cyp51, Hsd17b7), cholesterol esterification and transport regulators (Acat2, Stard4), negative regulation of cholesterol synthesis (Insig1), LDL‐cholesterol uptake (Ldlr), and fatty acid desaturation (Scd2); and Figure S3 illustrates the effects of 7KC and 7‐dehydrocholesterol (7DHC) on ferroptotic cell death, showing PI staining of AML12 cells treated for 24 h with erastin (20 µM), 7KC (20 µM), 7DHC (20 µM), or chenodeoxycholic acid (CdCA, 100 µM), with red‐stained cells representing dead or damaged cells and unstained cells representing viable cells.

## Data Availability

The data that support the findings of this study are available from the corresponding author upon reasonable request.
