# Supplementary material for: Hormetic Effects of 7‐Ketocholesterol in Preventing Ferroptosis in Hepatocytes
Source: Oxid Med Cell Longev. 2026 Feb 11;2026:7958511. doi: 10.1155/omcl/7958511 (PMC12892878; doi:10.1155/omcl/7958511)
Supplement: Supplementary file 1 — Supporting Information The supporting section describes additional analyses of 7KC effects on ferroptosis and lipid metabolism: Figure S1 shows the effect of 7KC on cell viability in ferroptotic hepatocytes, where AML12 cells were treated with 7KC and erastin for 24 h and viability was assessed by propidium iodide (PI) exclusion, with red‐stained cells indicating dead or damaged cells and unstained cells indicating viable cells; Figure S2 presents the effect of 7KC on cell viability in glutamate‐induced ferroptosis of HT4 neuronal cells, assessed by PI staining, including (A) dose‐dependent treatment with 7KC (10–50 µM) and (B) treatment with 10 mM glutamate in the presence of 10 or 20 µM 7KC for 18 h, with 10,000 cells counted per condition (n = 4) and statistically significant differences denoted by different letters (p < 0.05); Table S1 lists of differently affected genes by 7KC and erastin predominantly involved in cholesterol, lipid, and sterol metabolism, including key enzymes of the mevalonate pathway (Hmgcs1, Hmgcr), downstream isoprenoid‐processing enzymes (Mvd, Pmvk, Idi1), acetyl‐CoA–providing enzymes (Aacs, Acss2), sterol biosynthesis enzymes (Fdft1, Sqle, Lss, Nsdhl, Msmo1, Cyp51, Hsd17b7), cholesterol esterification and transport regulators (Acat2, Stard4), negative regulation of cholesterol synthesis (Insig1), LDL‐cholesterol uptake (Ldlr), and fatty acid desaturation (Scd2); and Figure S3 illustrates the effects of 7KC and 7‐dehydrocholesterol (7DHC) on ferroptotic cell death, showing PI staining of AML12 cells treated for 24 h with erastin (20 µM), 7KC (20 µM), 7DHC (20 µM), or chenodeoxycholic acid (CdCA, 100 µM), with red‐stained cells representing dead or damaged cells and unstained cells representing viable cells. [file OMCL-2026-7958511-s001.docx]

**Supplement:
Hormetic Effects of 7-Ketocholesterol in Preventing hepatocytes and neuronal cells Ferroptosis**

7KC

Ctrl


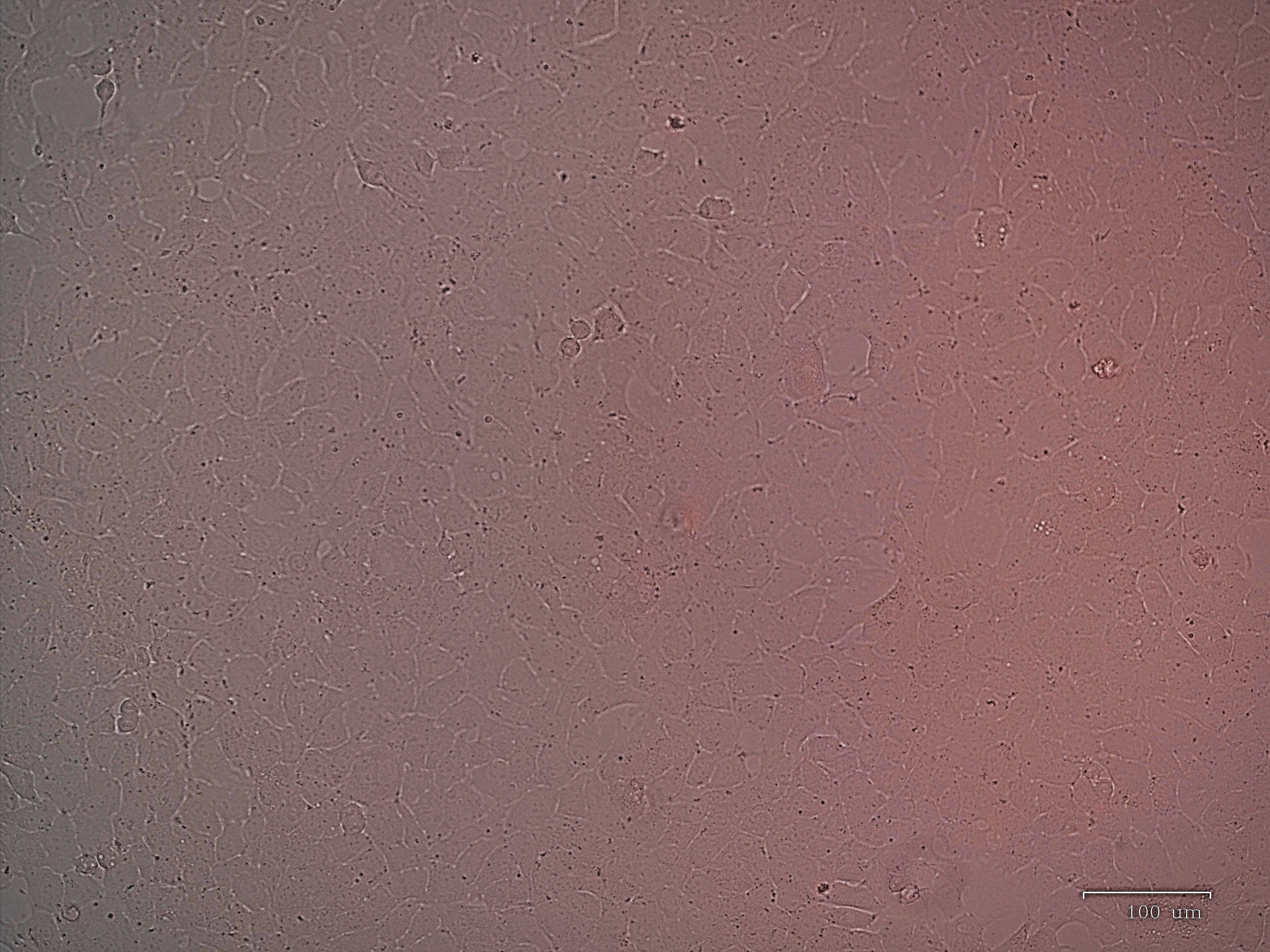

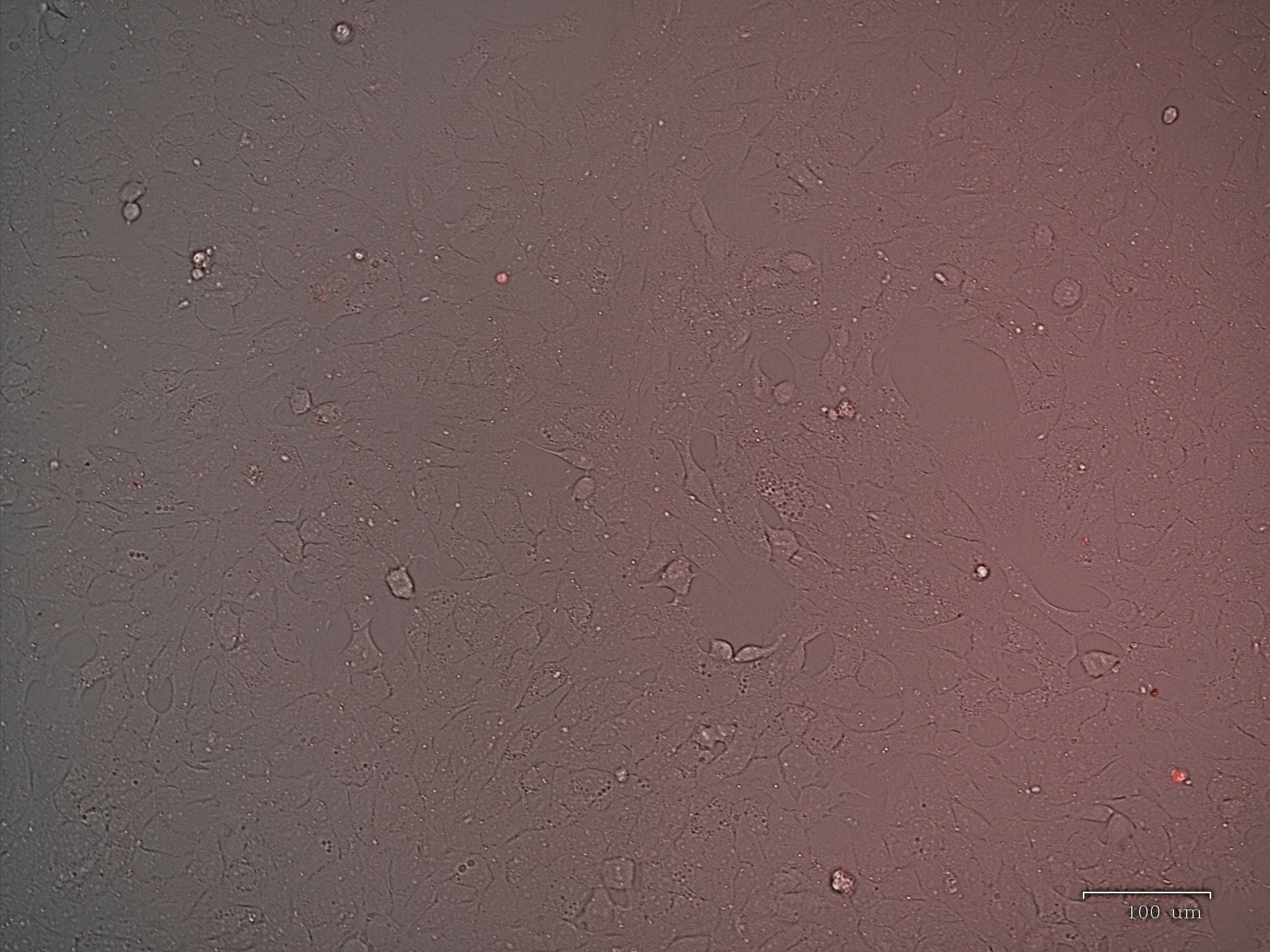

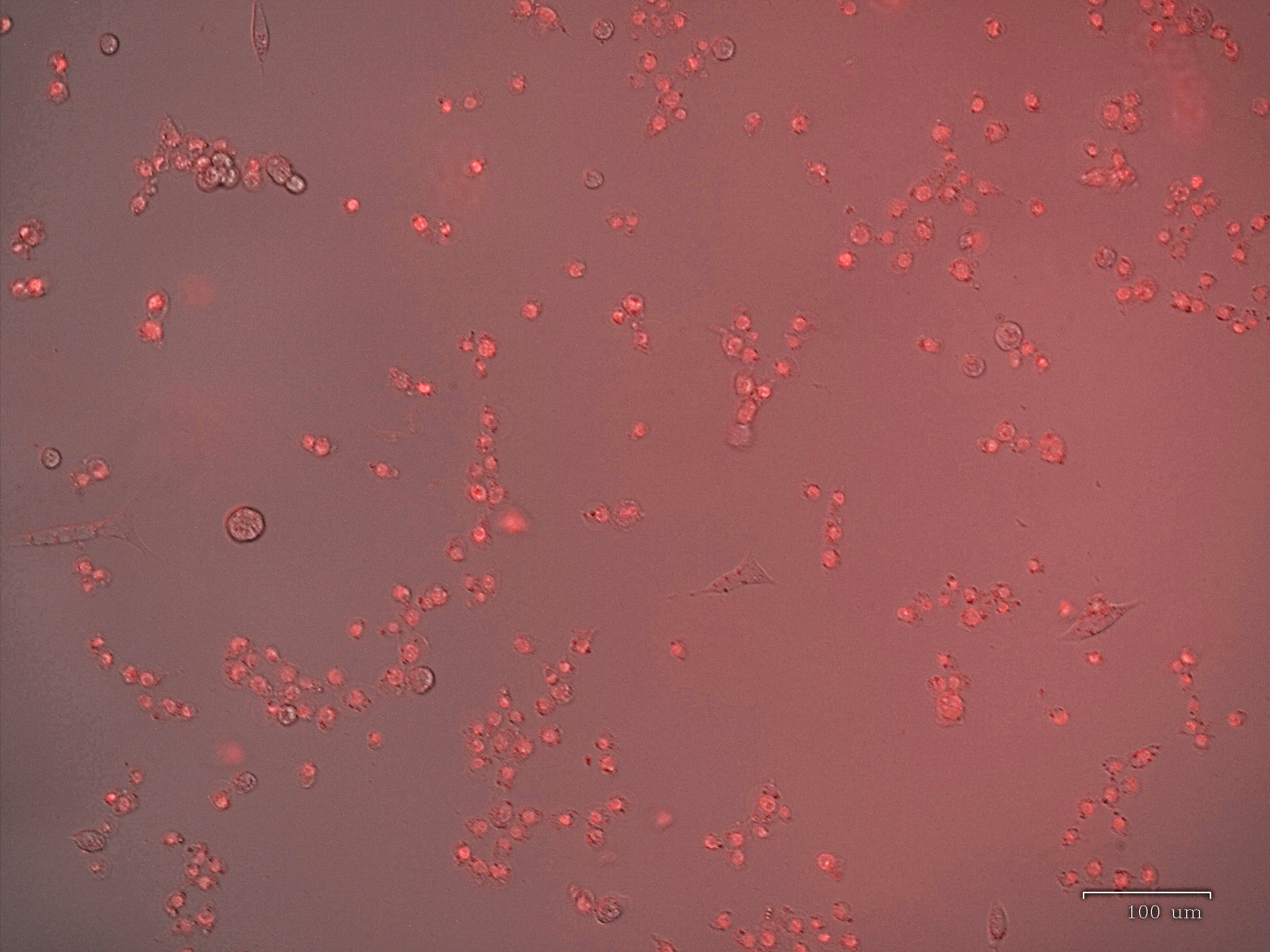

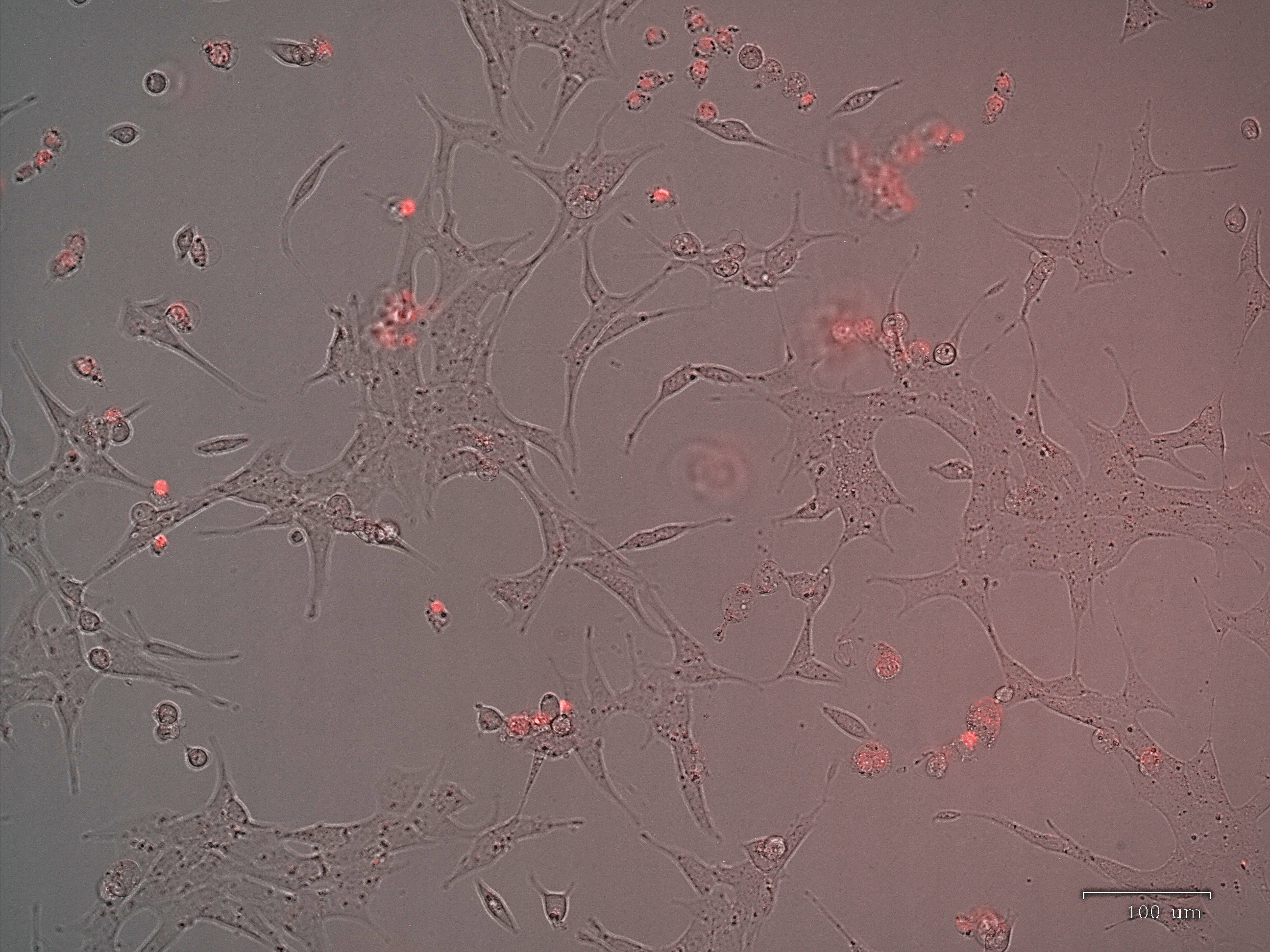


Vehicle

Erastin

**Figure 1S: The effect of 7KC on cell viability in ferroptosis hepatocytes**. AML12 cells viability was evaluated using the Propidium iodide (PI) exclusion method. PI staining of AML12 cells that were treated with 7KC and Erastin for 24 hours. Red-stained cells: dead/damaged cells; non-stained cells: live cells.

**Figure 2S:** **The effect of 7KC on cell viability in glutamate-induced ferroptosis of HT4 neuronal cells**. Cells were evaluated using the PI method. (A) viability of HT4 mouse hippocampal neuronal cells treated dose-dependently with 7KC (10, 20, 30, 40, 50 µM of 7KC). (B) viability of HT4 cells treated with 10mM glutamate, 10µM 7KC and 20µM 7KC, for 18 hours. (10,000 cells were counted). n = 4, Means with different letters are statistically different (p < 0.05).

Table 1S: Differentially expressed genes by RNAseq. The average was computed from the normalized counts

| Gene | Erastin (ave) | Control (ave) | 7KC  (ave) | 7KC + Erastin  (ave) | best__E7KCv7KC | best__E7KCvC | best__E7KCvE | best__EvC | best__KCvC |
| --- | --- | --- | --- | --- | --- | --- | --- | --- | --- |
| Cyp51 | 11624.73214 | 6071.168097 | 1653.384266 | 4864 | THR | FALSE | THR | THR | THR |
| MVD | 1036.758542 | 776.3371073 | 239.4700553 | 320 | FALSE | THR | THR | THR | THR |
| Lpin1 | 504.6341038 | 283.3852826 | 274.839002 | 208 | FALSE | TRUE | THR | FALSE | FALSE |
| Fdft1 | 5381.299125 | 3183.345341 | 761.2735165 | 1701.333333 | THR | THR | THR | THR | THR |
| Hmgcr | 5565.506525 | 4114.410709 | 1487.447843 | 2427 | TRUE | THR | THR | THR | THR |
| Sqle | 6768.17896 | 3921.718223 | 1161.124832 | 2888.333333 | THR | TRUE | THR | THR | THR |
| Acat2 | 1562.200754 | 1626.524589 | 654.0174326 | 680 | FALSE | THR | THR | THR | THR |
| Stard4 | 2126.492997 | 2362.912174 | 1454.0886 | 1011.666667 | TRUE | THR | THR | TRUE | TRUE |
| Scd2 | 52274.25331 | 35398.35527 | 27474.71732 | 27610.33333 | FALSE | FALSE | THR | FALSE | FALSE |
| Hsd17b7 | 2885.651926 | 1469.459373 | 853.6381595 | 1217.333333 | TRUE | FALSE | THR | THR | THR |
| Acss2 | 240.98222 | 118.0800442 | 90.84909981 | 121.6666667 | FALSE | FALSE | THR | FALSE | FALSE |
| Pmvk | 1051.425882 | 619.8898838 | 310.8960688 | 459.3333333 | FALSE | FALSE | THR | FALSE | FALSE |
| Aacs | 2587.628621 | 1560.986705 | 816.9405441 | 1214.666667 | FALSE | FALSE | THR | FALSE | FALSE |
| Nsdhl | 1874.426024 | 1715.102313 | 629.5745663 | 708.6666667 | FALSE | THR | THR | THR | THR |
| Msmo1 | 7871.776192 | 4315.91645 | 1265.577431 | 3885.333333 | THR | FALSE | THR | THR | THR |
| Ldlr | 10646.02675 | 4966.762091 | 1083.995472 | 4184 | THR | FALSE | THR | THR | THR |
| Lss | 5197.395671 | 1866.852325 | 688.1145151 | 2083.666667 | THR | FALSE | THR | THR | THR |
| Insig1 | 4346.529053 | 3024.290875 | 703.1741147 | 1513 | THR | THR | THR | THR | THR |
| Idi1 | 6369.418564 | 2659.503858 | 820.7748487 | 2014 | THR | FALSE | THR | THR | THR |
| Slc4a11 | 907.7972036 | 916.4480391 | 649.1401862 | 559 | FALSE | TRUE | TRUE | FALSE | FALSE |
| Hmgcs1 | 4665.931203 | 2580.580751 | 1066.976218 | 1736.333333 | FALSE | FALSE | THR | FALSE | FALSE |

The genes listed are largely involved in cholesterol, lipid, and sterol metabolism. **Hmgcs1** and **Hmgcr** are key enzymes in the mevalonate pathway; **MVD**, **Pmvk**, and **Idi1** process mevalonate derivatives to form essential isoprenoids; **Aacs** and **Acss2** provide acetyl-CoA and acetoacetyl-CoA for lipid and cholesterol synthesis. **Fdft1** initiates sterol formation by synthesizing squalene; **Sqle** and **Lss** convert squalene into lanosterol; **Nsdhl**, **Msmo1**, **Cyp51**, and **Hsd17b7** are involved in sequential demethylation and modification of sterol intermediates toward cholesterol formation; **Acat2** participates in cholesterol esterification and lipid absorption; **Stard4** transports cholesterol between intracellular membranes; **Insig1** negatively regulates cholesterol synthesis by retaining the SREBP-SCAP complex in the endoplasmic reticulum; **Ldlr** LDL-cholesterol uptake from the blood; **Scd2** catalyzes the formation of monounsaturated fatty acids; **Lpin1** regulates triglyceride and phospholipid synthesis while also acting as a co-regulator of metabolic genes; **Hsd17b7** additionally participates in steroid metabolism; **Slc4a11** ion and water transporter involved in cellular homeostasis.


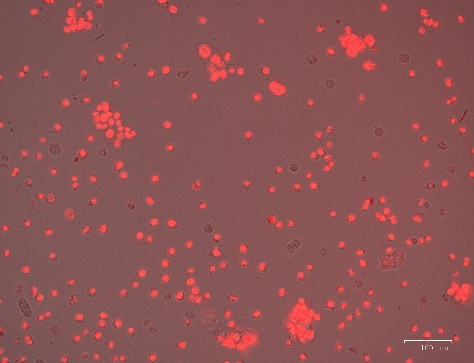

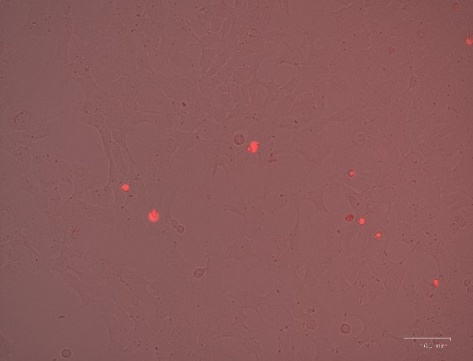

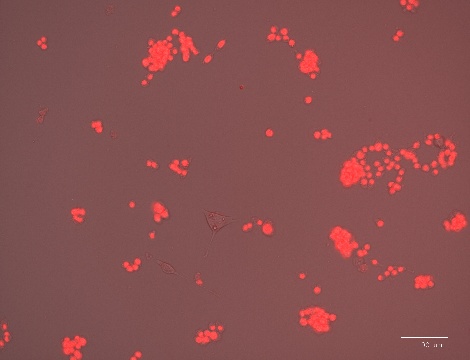

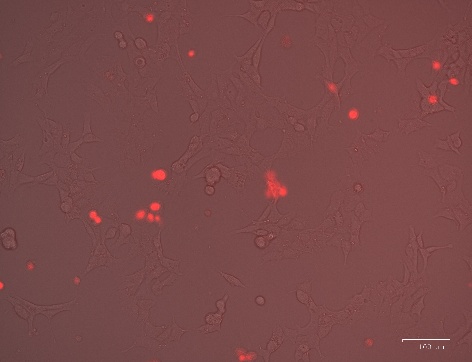

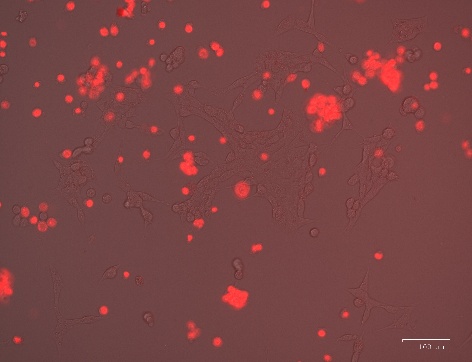

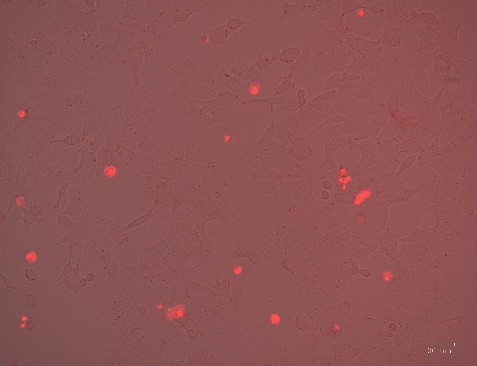

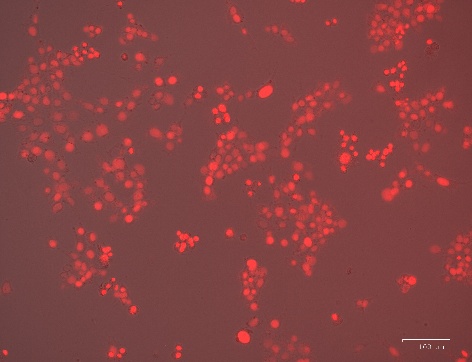

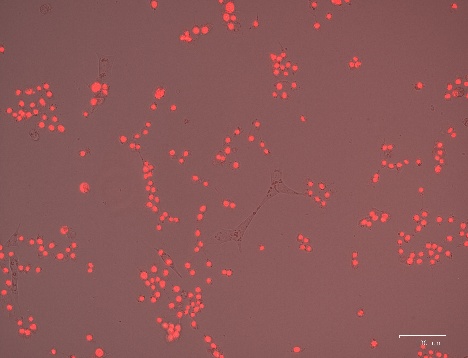

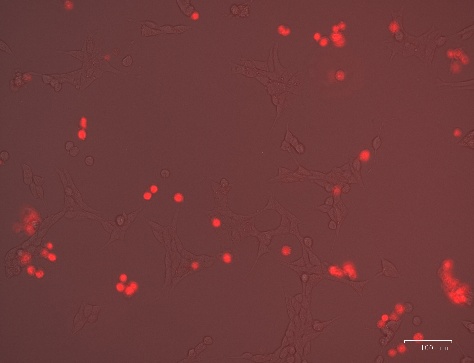


Ctrl

7KC

7-DHC

Vehicle

Erastin

CdCa+
Erastin

e

**Figure 3S: The effect of 7KC and 7DHC on ferroptosis cell death**. PI staining of AML12 treated with 20µM Erastin, 20µM 7KC, 20µM 7-DHC, 100µM Chenodeoxy Cholic Acid (CdCA) for 24 hours. Red-stained cells: dead/damaged cells; non-stained cells: live cells.
